# Development and validation of artificial intelligence-based full dentition labiolingual reconstruction using CBCT images

**DOI:** 10.1186/s12903-026-09611-w

**Published:** 2026-08-19

**Authors:** Xiao Pan, Wenxi Zhang, Xuhui Luo, Wentao Zhang, Hongyan Qu, Runzhi Deng, Zitong Lin

**Affiliations:** 1 Department of Dentomaxillofacial Radiology, Nanjing Stomatological Hospital, Affiliated Hospital of Medical School, Institute of Stomatology, Nanjing University, Zhong Yang Road 30, Nanjing, Jiangsu Province 210008 P.R. China; 2 Department of Preventive Dentistry, Nanjing Stomatological Hospital, Affiliated Hospital of Medical School, Institute of Stomatology, Nanjing University, Nanjing, China; 3 Shanghai Bondent Technology Co., Ltd., Shanghai, 201800 China; 4 Department of General Dentistry, Nanjing Stomatological Hospital, Affiliated Hospital of Medical School, Institute of Stomatology, Nanjing University, Zhong Yang Road 30, Nanjing, 210008 P.R. China

**Keywords:** Artificial intelligence, Cone beam computed tomography, Full dentition labiolingual reconstruction

## Abstract

**Objective:**

To development and validate an artificial intelligence (AI) -based system for automatic full dentition labiolingual reconstruction (DenRec-LL) using Cone beam computed tomography (CBCT) images.

**Methods:**

DenRec-LL was developed by generating 2D panoramic images, segmenting and identifying teeth with a transformer framework, mapping tooth centroids into 3D coordinates for 2D–3D alignment, performing 3D tooth segmentation using deep learning, and reconstructing the full dentition labiolingually based on estimated tooth axes, centers, and missing-tooth prediction. 75 external CBCT datasets (25 normal dentition, 25 mixed dentition and 25 crowded dentition) were collected and used for testing. The performance of DenRec-LL system was evaluated on a 4-point scale, the geometric deviation metrics were analyzed and the clinical acceptability rate (CAR) was calculated, and the reconstruction time was recorded.

**Results:**

75 CBCT datasets with 1500 teeth demonstrates high precision and stability across normal, mixed, and crowded dentition. A clear majority of labiolingual reconstruction cases (93.1%) achieved the highest score, and CAR was approximately 94% across groups, with no significant differences. The automated process required 63 ± 4 s per case, representing an approximately 4.85-fold reduction compared to the manual process.

**Conclusions:**

The DenRec-LL system could be used for full dentition reconstruction with high accuracy, robustness and efficiency.

**Clinical significance:**

DenRec-LL bridges the gap between raw CBCT data and clinical decision‑making in routine orthodontic practice by automatically generating full‑dentition labiolingual images, which facilitate the assessment of root‑bone relationships and torque control.

## Introduction

Cone beam computed tomography (CBCT), which offers high spatial resolution and low radiation exposure, has been widely used across multiple disciplines in dentistry [1]. A CBCT dataset typically consists of several hundred axial images, which provide details of each tooth in the horizontal plane. As a three-dimensional dataset, it is also essential to evaluate the teeth from mesiodistal and buccolingual views. In clinical practice, a comprehensive and intuitive assessment of the entire dentition often requires a curved planar mesiodistal reconstruction (similar to a panoramic radiograph) and labiolingual/buccolingual reconstructions of teeth of interest. However, such reconstructions are challenging due to several factors:1) the dentition is arranged in a curved arch within the jawbones; 2) the morphology of dental arch varies among patients, and 3) the three-dimensional orientation of each tooth is distinct. Consequently, a whole reconstruction of the maxillary and mandibular dentition using CBCT images remains a quite complicated and time-consuming task.

The development of AI systems in oral radiology lags that in medical radiology, where AI applications such as pulmonary nodule screening and coronary function assessment have been developed and routinely used in clinical practice for several years. Until recently, deep learning‑based segmentation of the mandible, maxilla, and dentition achieved rapid progress [2–10], focusing either on specific anatomical structures (such as mandible, maxillary or the mandibular nerve canal, et al. [11–15]) or segmenting and identification the dentition using panoramic radiograph or CBCT dataset [7, 9, 10, 13, 16]. Meanwhile, AI-assisted three-dimensional tooth reconstruction, and AI-assisted multimodal combining of CBCT with intraoral scanning have also been reported. The former focuses mainly on preoperative surgical planning for dental implants, while the latter is used for automatic registration of CBCT and intraoral scanning data to obtain high-fidelity 3D tooth models, as well as for implant planning and periodontal disease diagnosis, et al. [17–23]. In orthodontics, the labiolingual/buccolingual reconstruction slices are helpful for assessment of root-bone relationships and torque control. However, the manual full dentition reconstruction is quite time-consuming and labor-intensive. However, no AI studies have explored how to automatically generate labiolingual or buccolingual reconstructed slices from CBCT images.

In this study, to solve the complicated and time-consuming reconstruction in clinic, we develop an automatic tooth reconstruction system for full dentition using CBCT images. The innovation of our system included: 1) It proposes a novel bidirectional 2D–3D integration framework, enabling effectively combining the computational efficiency of 2D processing with the spatial precision of 3D volumetric analysis. 2) Building on efficient automatic segmentation and identification of the entire dentition, we developed a model that identifies each tooth's long axis by calculating its spatial center and orientation. 3)Based on teeth segmentation and long axis estimation, our system automatically generates labiolingual reconstruction images for the full dentition, providing superior morphological presentation compared to conventional standard coronal or sagittal views. This study proposed artificial intelligence-based full dentition labiolingual reconstruction, making the application of artificial intelligence using CBCT images more practical and responsive to real-world clinical demands.

## Materials and methods

### DenRec-LL system development

**Fig. 1:**
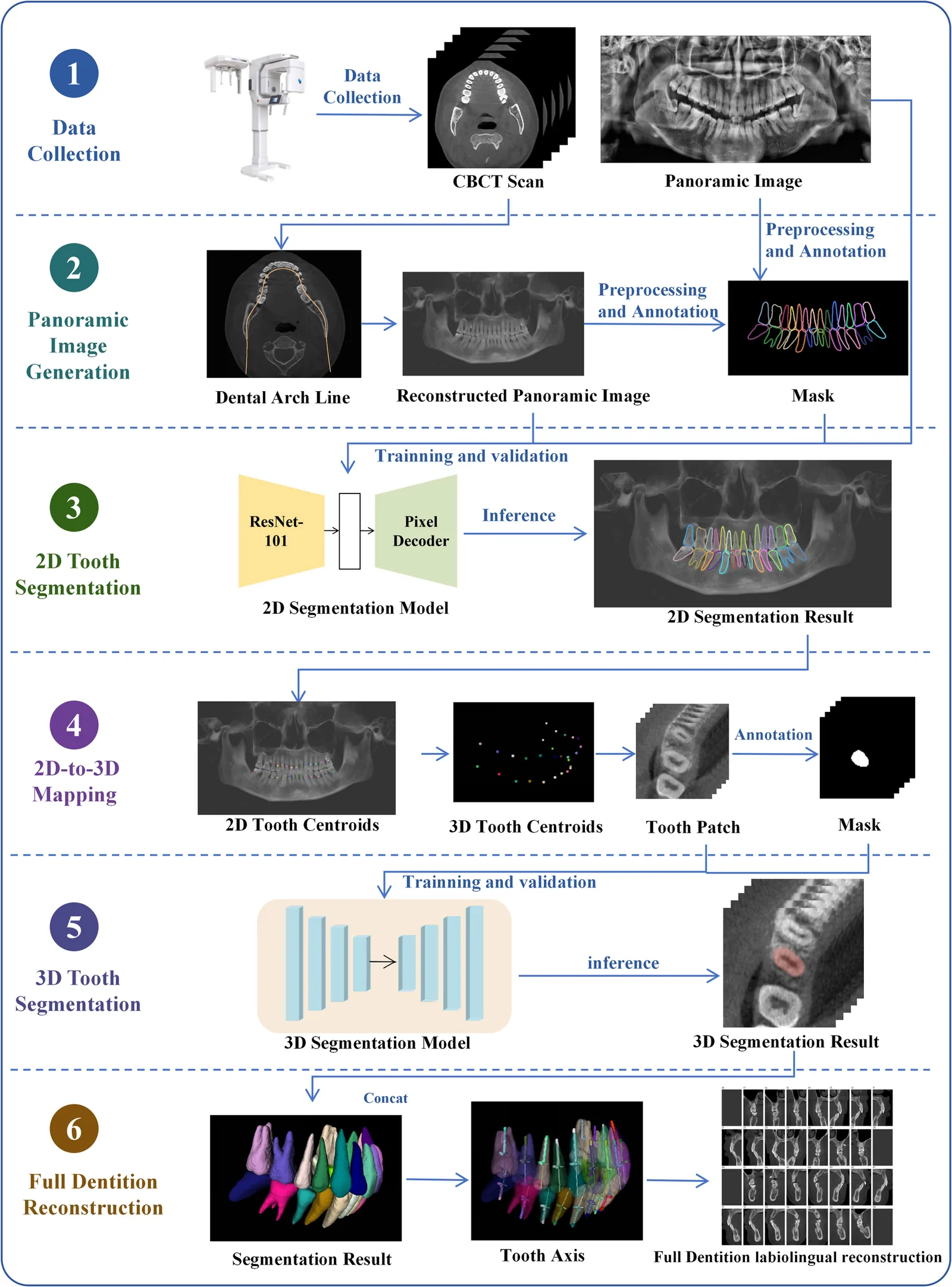
Overall architecture of the DenRec-LL system

The workflow of this DenRec-LL system was showed in Fig. 1.

#### Data collection

160 CBCT scans (160 patients) were collected at Nanjing Stomatological Hospital for 3D model development. These scans contained approximately 5,100 individual teeth, which were used as samples for 3D tooth segmentation. In addition, 3,000 panoramic images from 3,000 patients were collected for 2D tooth segmentation, including 2,000 clinically captured panoramic images and 1,000 panoramic images reconstructed from CBCT volumes, ensuring consistency between imaging modalities.

All CBCT scans and panoramic images were captured using Bondream 3D-1040 devices (Bondent, Shanghai, China). The voxel sizes of CBCT scans ranged from 0.20 mm to 0.30 mm. The inclusion criteria were as follows: (1) high-quality CBCT images without significant motion or metal artifacts; (2) the maxillary and mandibular dentition and alveolar bone structures were complete presented in the images (3) patient age between 10 and 80 years, with randomized gender distribution and no restriction on dental pathology type.

All data were anonymized before analysis. Two certified dental radiologists independently annotated individual tooth contours and tooth IDs on both 2D and 3D datasets using professional imaging software. Any discrepancies in annotation were resolved by consensus review.

#### 2D panoramic image automated generation

To efficiently capture the overall dental geometry while reducing computational complexity, 2D panoramic images were reconstructed from 3D CBCT scans.

The dental arch curve was extracted and interpolated so that the distance between adjacent sampling points equaled one pixel. For each point along the dental arch, a perpendicular bisector line was determined to represent the direction orthogonal to the tooth alignment. Along this direction, voxel intensities from the CBCT slices were sampled and integrated to form the corresponding pixel in the panoramic projection. Iterating this process along the entire arch produced a complete panoramic image that preserved the anatomical continuity of the dentition.

#### 2D tooth segmentation model

A transformer-based segmentation framework, Mask2Former, was employed to achieve tooth segmentation and ID classification from panoramic images. The network integrates a ResNet-101 backbone for multi-scale feature extraction, a pixel decoder for high-resolution reconstruction, and a transformer mask decoder that generates per-tooth segmentation masks and corresponding ID predictions.

All images were resized to and normalized to zero mean and unit variance. The model achieved an Average Precision (AP) of 90.55% and an AP@0.50 of 99.23% on the validation set.

The purpose of this stage was to identify each tooth and obtain its location on the panoramic image. These results were subsequently used to locate the corresponding tooth region in the original 3D CBCT scan.

#### 2D-to-3D mapping of tooth centroids

For each segmented tooth, a 2D centroid was computed from its contour. Using the geometric calibration between panoramic image and CBCT scan, each 2D centroid was projected along the dental arch’s perpendicular direction to locate its 3D centroid coordinate. A cubic patch of $96\times 96\times 96$ voxels centered at the mapped centroid was then cropped from the CBCT scan. If the patch exceeded image boundaries, padding with −1000 HU was applied.

This mapping procedure transforms the approximate tooth location detected in the panoramic image into a localized 3D region. It allows the subsequent model to focus on one tooth at a time and reduces interference from neighboring teeth and other anatomical structures.

#### 3D tooth segmentation model

A 3D deep learning framework based on 3D-UNet was employed to perform volumetric segmentation of individual teeth from CBCT scans.

The dataset comprised 160 CBCT volumes, containing approximately 5,100 individual teeth with expert-labeled tooth masks. Each tooth region was obtained from Section "2D-to-3D mapping of tooth centroids".

The model followed a self-configuring encoder-decoder architecture with skip connections for precise feature fusion.

The optimization objective combined Dice loss and BCE loss. Dice loss evaluates region overlap between prediction and ground truth, while BCE loss measures voxel-wise prediction accuracy.

The model was trained for 1500 epochs. At convergence, the training loss reached − 0.8191, while the validation loss was − 0.7999. The learning rate at the final stage of training was 1e-05. The model achieved a Pseudo Dice coefficient of 0.9514 on the validation set, with the best exponential moving average (EMA) Pseudo Dice reaching 0.947.

After segmentation, for each tooth, the corresponding centroid coordinate was used to compute its position in the original CBCT coordinate system. Overlapping voxels were resolved by retaining the maximum intensity value. All teeth were then merged to generate a complete 3D dentition segmentation.

#### Tooth axis estimation and full dentition reconstruction

After obtaining the individual 3D tooth segmentation results, each tooth’s spatial center and orientation were calculated to enable accurate full-dentition reconstruction. The process included three main stages: (1) tooth axis estimation, (2) prediction of missing teeth, and (3) full dentition reconstruction based on positional and directional information.

##### Tooth axis estimation

For each segmented tooth, voxel coordinate was extracted and centroids of axial slices were computed. The tooth center was defined as the centroid on the slice corresponding to the median Z-value. The tooth’s long axis was determined by fitting all slice-wise centroids using least-squares linear regression.

Cubic spline interpolation of the ordered tooth centers was applied to generate smooth dental arch curves for each jaw. The intersection between each tooth crown projection and the arch curve defined the mesiodistal direction. The labiolingual direction was obtained as the cross product of the long and mesiodistal axes.

##### Prediction of missing teeth

The center point of the missing tooth can be calculated as follows:

$$n=\frac{({n}_{2}-{n}_{1})({m}_{1}+1)}{{m}_{1}+{m}_{2}+2}+{n}_{1}$$

where $\mathrm{n}$ is the index of the missing tooth’s center point on the dental arch line, ${\mathrm{n}}_{1}$ and ${\mathrm{n}}_{2}$ are the indexes of the center point of the left and right adjacent natural teeth, respectively, ${\mathrm{m}}_{1}$ and ${\mathrm{m}}_{2}$ represent the numbers of missing teeth between the missing tooth and its left and right adjacent teeth.

The long axis direction of the missing tooth was estimated by linear interpolation between the long axis vectors of the nearest natural teeth on both sides. The interpolation weights were determined by the relative tooth position intervals, such that the long axis vector of the closer adjacent tooth contributed more strongly to the estimated direction. The resulting vector was subsequently normalized to obtain a unit direction vector. The mesiodistal direction was derived from the tangent of the arch curve. The labiolingual direction was computed by the cross product of the long and mesiodistal axes.

##### Full dentition reconstruction

With the estimated centers and axes of all teeth, the full 3D dentition was reconstructed. Taking the labiolingual longitude‌-sectional view as an example, the mapping between 2D slice coordinates (r, c) and 3D CBCT coordinates was expressed as:

$$\begin{aligned}{\mathbf{L}\mathbf{o}\mathbf{c}}_{3D}=&\mathbf{l}\mathbf{o}\mathbf{c}+(r-{r}_{0}){\mathbf{Z}}_{axis}\\+&(c-{c}_{0}){\mathbf{v}}_{LL}+t\hspace{0.17em}{\mathbf{v}}_{MD}\end{aligned}$$

where $\mathbf{l}\mathbf{o}\mathbf{c}$ is the 3D tooth center, $({r}_{0},{c}_{0})$ is the center of the reconstructed image, ${\mathbf{Z}}_{axis}$ is the global vertical direction, and ${\mathbf{v}}_{MD}$ are the labiolingual and mesiodistal direction of the tooth, and *t* is the reconstruction depth. Thus, each 2D pixel was mapped to the original CBCT scan according to the tooth center and its anatomical orientation.

Thus, each 2D pixel was mapped to the original CBCT scan according to the tooth center and anatomical orientation. The tooth slice was generated by averaging the corresponding CBCT voxel intensities across a predefined reconstruction thickness.

By combining the reconstructed tooth slices, the system generated a complete digital model of the dentition with anatomically consistent alignment and orientation.

### External testing and model performance evaluation

#### CBCT data collection

CBCT scans of patients who underwent examination between October 2023 and October 2024 in Nanjing Stomatological Hospital were retrospectively collected. All the CBCT scans were taken using NewTom VGi(Qualitative Radiology SRL Co.,Ltd, Verona, Italy). The inclusion criteria were as follows:(1) patient age between 6 and 30 years, with randomized gender distribution and no restriction on dental pathology type; (2) the maxillary and mandibular dentition and alveolar bone structures were complete presented in the CBCT images; (3) CBCT images without severe motion or metal artifacts; (4) no missing teeth in the regions from teeth 15 to 25, 35 to 45. The CBCT data were categorized into three groups based on dentition characteristics: normal dentition group (Group A), a mixed dentition group (Group B), and crowded dentition group (Group C). The Group A and C were both permanent dentitions. On CBCT axial images, if ≥ 1 tooth arranged away from the dental arch or if ≥ 1 tooth with rotation ≥ 45°, the dentition is defined as crowded dentition. Ultimately, 25 CBCT datasets meeting the criteria were included in each group, resulting in a total of 75 CBCT datasets for external testing.

All CBCT data were exported in Digital Imaging Communications in Medicine (DICOM) format and fully anonymized. Prior to conducting this study, approval was obtained from the Ethics Committee of Nanjing Stomatological Hospital, Medical School of Nanjing University [Ethics Approval: NJSH-2025NL-043]. To protect patient privacy, we strictly comply with data privacy and protection laws and regulations, and have undergone strict anonymization of all patient data during the collection process. Due to the use of de- identified historical medical records in this study and the fact that the study protocol posed no additional risks to participants, the ethics committee waived informed consent, in compliance with the “Guidelines for the Construction of Ethical Review Committees for Clinical Research Involving People” (2019).

#### Establishment of gold standard

All CBCT images included in this study were independently reconstructed by two oral and maxillofacial radiologists with five years’ experience (Evaluator A and Evaluator B) using the DenRec-LL system. When the images reconstructed by the two evaluators were visually similar, the images from Evaluator A were adopted as the ground truth. For teeth with significant inter‑evaluator differences, a third senior radiologist with 10 years of experience performed a second reconstruction, and this result was taken as the ground truth. Three months later, Evaluator A performed a manual reconstruction of these teeth once again to calculate intra-assessor reproducibility. Prior to the manual reconstruction, all three evaluators underwent standardized training on the reconstruction process: firstly, find the axial image at the level of the cemento-enamel junction, then find the line connecting the midpoints of the mesial and distal margins of the tooth, and finally perform the reconstruction along the direction perpendicular to this line.

#### Scoring of automatic tooth identification and labiolingual reconstruction

**Fig. 2:**
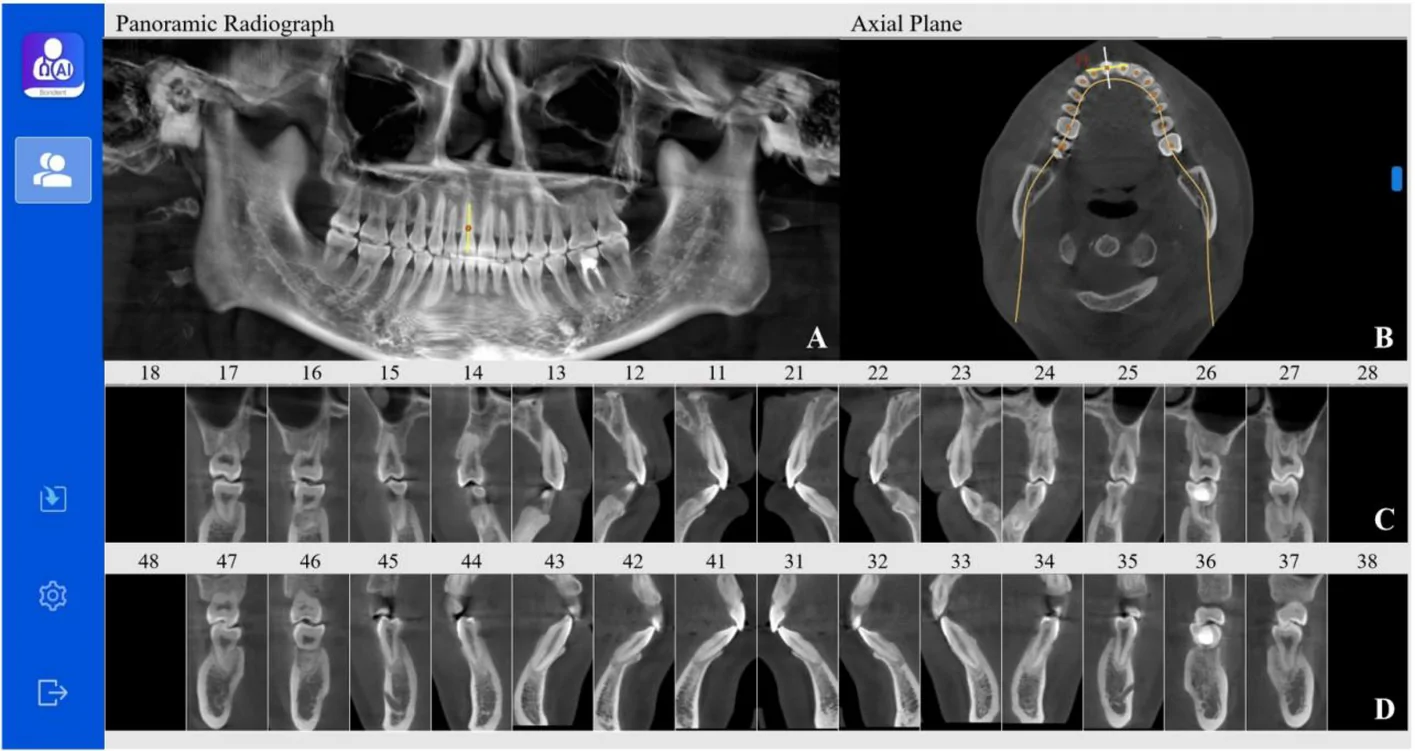
The interface of DenRec-LL system: **A** panoramic reconstructed image using the CBCT data, for tooth 11, the yellow line is the long axis of the tooth and the red point is the centroid; **B** The axial image showing the dental arch curve (the orange line) and the centroid of each tooth(the red point), the yellow line is the mesiodistal proximal midpoint line, the white line is the labiolingual reconstruction plane. **C** Labiolingual reconstruction view of the maxillary dentition. **D** Labiolingual reconstruction view of the mandibular dentition

**Fig. 3:**
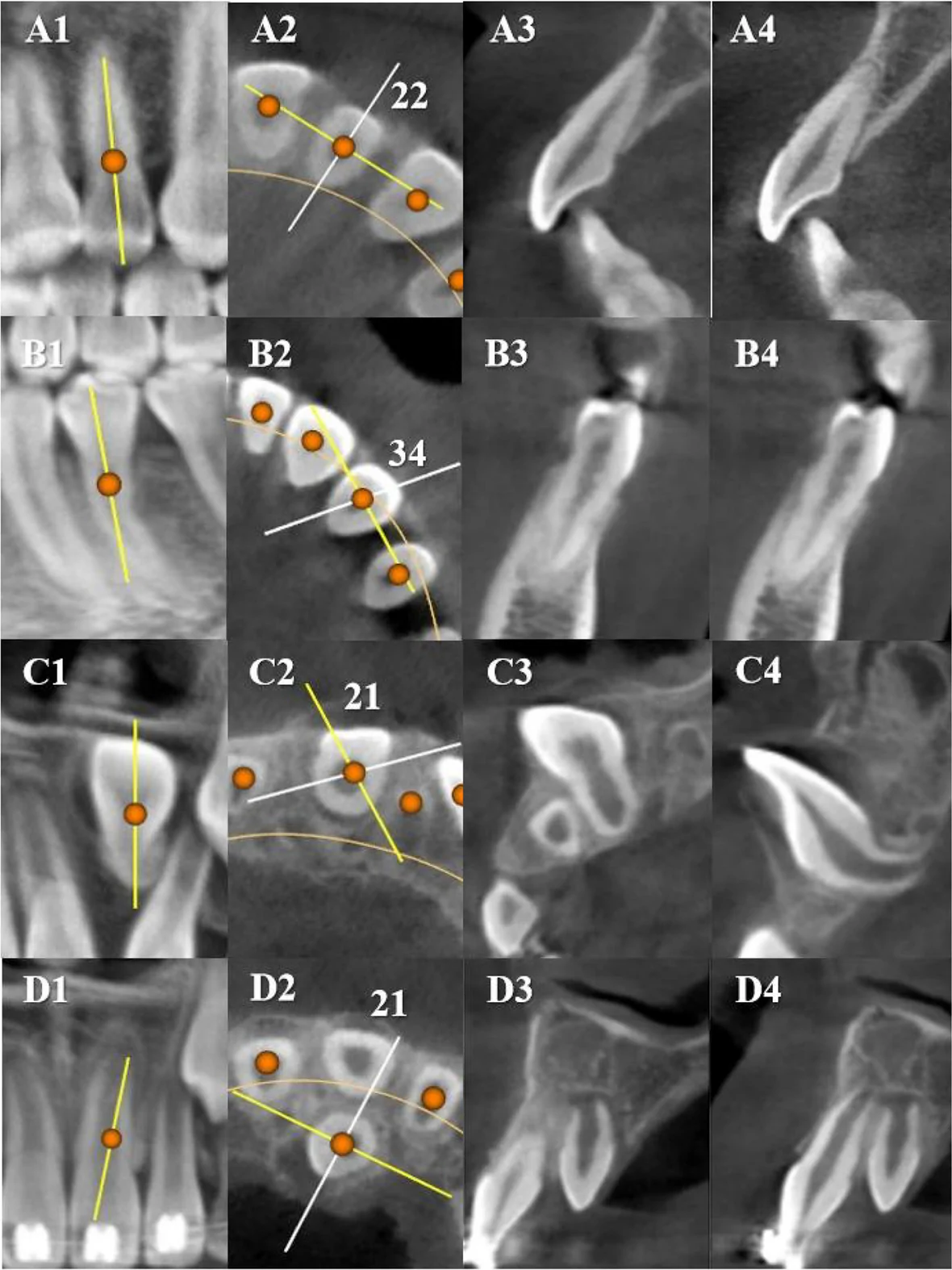
Representative reconstructed images scoring 3–0 point: **A1**-**D1** Panoramic reconstruction images showing the long-axis of the target tooth(yellow line); **A2**-**D2** axial images showing the labiolingual reconstruction plane; **A3**-**D3** Automated labiolingual reconstructed images; **A4**-**D4** Manual labiolingual reconstructed images. **A1**-**A3** showing tooth 22 scoring 3 points, accurate tooth identification, accurate tooth axis positioning, and complete crown-to-root labiolingual reconstruction, the manual reconstructed image is quite the same with the AI; **B1**-**B3** showing tooth 34 scoring 2 points, accurate tooth identification, accurate tooth axis positioning, but the root was not complete in the labiolingual reconstructed image, **B4** showing the manual reconstructed image with complete crown-to-root; **C1**-**C3** showing tooth 21 scoring 1 point, accurate tooth identification, but **C2** showing the tooth axis(the orange point) and the labiolingual reconstruction plane(the yellow line) are not accurate, **C3** showing a poor reconstructed image, **C4** showing the manual reconstructed image with complete crown-to-root; **D1**-**D3** showing tooth 21 scoring 0 point, the tooth identification is wrong, **D2** showing the tooth axis(the orange point) and the labiolingual reconstruction plane are in the supernumerary tooth but not 21, **D3** showing a poor reconstructed image, **D4** showing the manual reconstructed image of 21 with complete crown-to-root

The CBCT images of 75 patients were imported into the DenRec-LL platform for automated tooth identification, segmentation and reconstruction (Fig. 2). In this study, the AI system automatically generated labiolingual slices for the maxillary and mandibular dentition. For multi-root molars, the tooth axis was determined by minimizing its distance to all roots. As a result, it was sometimes impossible for the labiolingual reconstructed images to fully capture the crown along with any one complete root for multi-root molars (Fig. 2). Therefore, although labiolingual images were generated for the full dentition by the DenRec-LL system, only teeth 15–25 and 35–45 were scored in this study. This excluded the multi-root permanent molars in Groups A and C. For Group B, scoring was based on these same permanent teeth, or on the reconstructed tooth germ if the permanent tooth was unerupted. A 4-point Rating scale (Table 1) was used to evaluate the performance of this AI system for tooth identification and dentition reconstruction (Fig. 3). The scoring was performed by two radiologists who were blinded to patient grouping. In cases of scoring disagreement, the images were re-evaluated by a third senior radiologist, whose score was considered final.

**Table 1:** Scoring for full dentition labiolingual reconstruction

Score	Definition
3	Accurate tooth identification, accurate tooth axis positioning, and complete crown-to-root labiolingual reconstruction
2	Accurate tooth identification, but with a deviation in tooth axis positioning or labiolingual reconstruction
1	Accurate tooth identification, but with deviations in both tooth axis positioning and labiolingual reconstruction
0	Incorrect tooth identification

#### Geometric deviation metrics analysis

For the teeth scoring 0–2, the geometric deviation metrics were analyzed. The geometric parameters of automatic reconstruction and the manual reconstruction were recorded: (1) 3D coordinates of the plane center point (X, Y, Z, in mm, based on the DICOM origin); (2) plane normal vector (a, b, c, as a unit vector). Additionally, to describe the spatial orientation of teeth, three sets of orthogonal direction vectors were recorded: long axis direction, along-arch direction, and to-midline direction.

The model used in this study automatically generates plane parameters for each tooth, including the coordinates of the center point and the normal vector, as well as three sets of direction vectors. The model’s output format is fully consistent with the ground truth annotations.

Based on the comparison between AI-predicted parameters and ground truth parameters, the following metrics were calculated:

- (1) Angular Deviation (AD): Angular deviations were calculated for the three direction vectors (long axis, along-arch, to-midline) and the plane normal vector.
- (2) Distance Deviation (DD): The Euclidean distance between the AI-predicted center point and the ground truth center point was calculated.
- (3) Clinical Acceptance Rate (CAR): Based on clinical practice requirements, an acceptable plane was defined as one satisfying both plane angular deviation < 5° and center point distance deviation < 0.5 mm [18, 24, 25]. CAR was calculated as the percentage of teeth meeting these criteria out of the total number of teeth.

#### Time efficiency

To evaluate time efficiency, three types of reconstruction time were recorded for 10 randomly selected CBCT datasets from each group: (1) manual reconstruction time: the total time of manually reconstruction for teeth 15–25 and 35–45 (reconstruction time of 20 teeth); (2) automatic reconstruction time: the time of full dentition reconstruction performed by the AI system; (3) automatic reconstruction + manual adjustment time: full dentition reconstruction by the AI system + manual adjustment of teeth that need correction in teeth 15–25 and 35–45.

### Statistical analyses

SPSS23.0 software (IBM Corp., Armonk, NY, USA) was used for the statistical analysis. For the quantitative data, values were presented as the mean ± standard deviation (SD). The proportion of scores for each group was calculated, and chi-square tests were performed to compare the differences among the three groups. The significance level for all statistical tests was set at α = 0.05, meaning that differences were considered statistically significant when the p-value was less than 0.05. Inter-evaluator and intra-evaluator reproducibility for manual reconstruction was assessed using the intraclass correlation coefficient (ICC) with a two-way random-effects model for absolute agreement.

## Results

### Full dentition labiolingual reconstruction scoring

The full dentition labiolingual reconstruction scoring of the normal dentition, mixed dentition and crowded dentition were presented in Table 2. The vast majority of automatically generated labiolingual images (93.1%) received the highest score of 3 points, indicating correct tooth identification, accurate tooth axis positioning, and complete crown-to-root labiolingual reconstruction. The chi-square test (Score0-2 vs. Score 3) showed no statistically significant differences among the three groups (*P* = 0.77), demonstrating stable and robust performance across varying dentition.

**Table 2:** The labiolingual reconstruction scoring of the three groups (*N* = 1500)

Group	Score 0	Score 1	Score 2	Score 3	*P*
Normal dentition	0(0.0%)	0(0.0%)	37(7.4%)	463(92.6%)	0.77
Mixed dentition	3(0.6%)	4(0.8%)	24(4.8%)	469(93.8%)	
Crowded dentition	2(0.4%)	0(0.0%)	33(6.6%)	465(93.0%)	
Total	5(0.3%)	4(0.8%)	94(6.3%)	1397(93.1%)	

The *P* value was the result of chi-square test between Score 0–2 and Score 3

### Geometric deviation metrics

For the 103 teeth scoring 0–2 in this study, the geometric deviation metrics showed the distance deviation of reconstruction center point was significantly larger in the mixed dentition group compared to the other two groups (Table 3). The clinical acceptability rate was 94.20% in the normal dentition group, 94.80% in the mixed dentition group, and 93.80% in the crowded dentition group. There was no statistically significant difference in clinical acceptability rates between the three groups (*p* > 0.05).

**Table 3:** Geometric Deviation Metrics of Teeth Scoring 0–2 (Mean ± SD)

Group (No.)	AD (°) long axis direction	AD (°) along-arch direction	AD (°) to-midline direction	AD (°) reconstruction plane	DD (mm) reconstruction center point
Normal dentition (37)	0	9.2 ± 4.9	8.8 ± 4.9	8.5 ± 4.8	0.1 ± 0.4
Mixed dentition (31)	6.4 ± 31	14.2 ± 15.6	14.5 ± 18.9	13.7 ± 18	5.3 ± 11.3
Crowded dentition (35)	0	11.9 ± 9.3	11.9 ± 9.4	11.6 ± 9.3	0.4 ± 1.6
P	-	0.10	0.08	0.09	0.03*

*AD* Angular Deviation*, DD* Distance Deviation

**Fig. 4:**
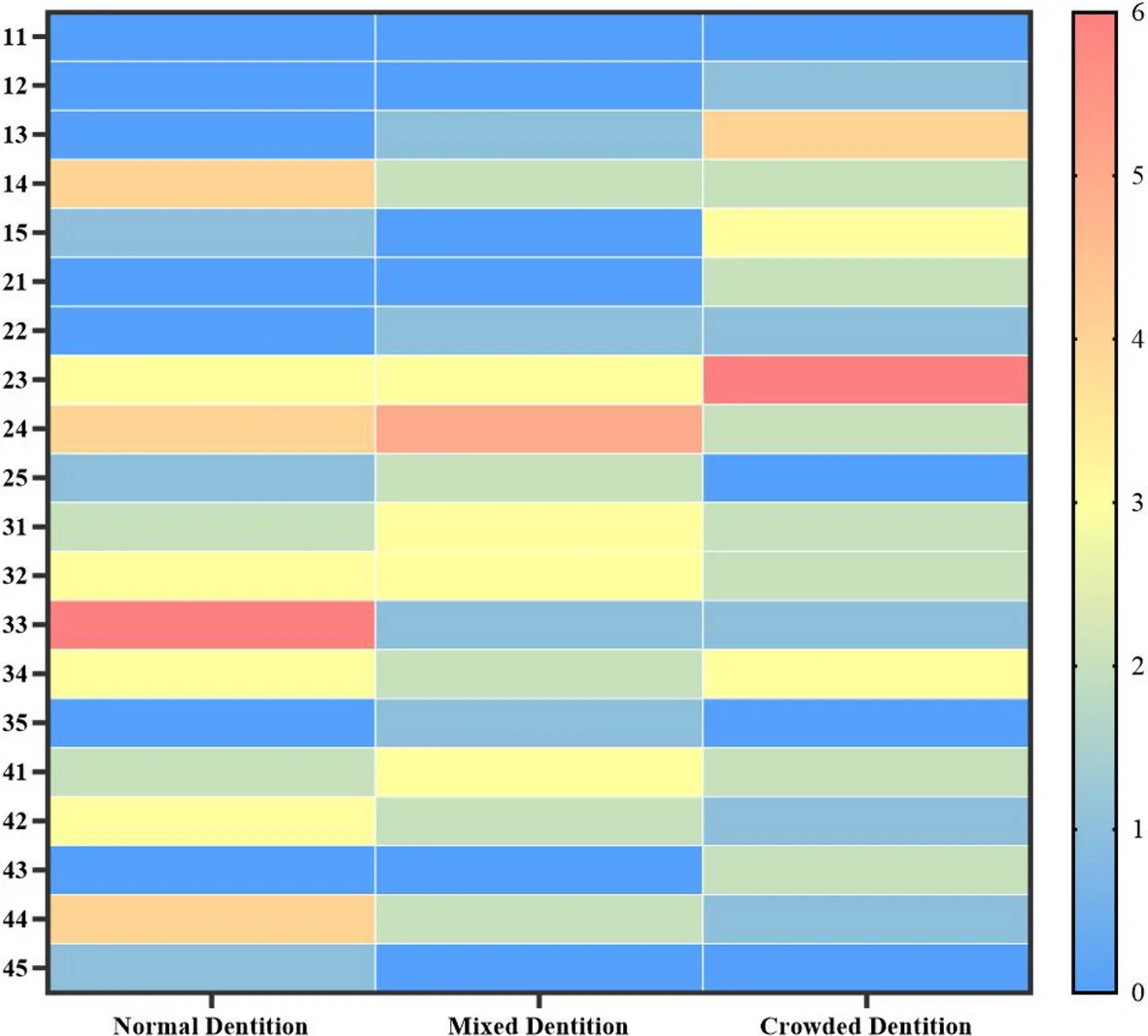
Distribution of low‑scoring teeth (scores 0–2) by position across the three groups, shown as a blue‑to‑red gradient where red indicates higher counts and blue lower counts

Statistics based on different tooth positions show that low-scoring teeth are more frequently observed in canines and first premolars (Fig. 4), and these teeth also exhibit relatively large angular deviations. Specifically, canines (angle deviation: tooth 13: 47.2–49.5°; tooth 23: 7.4–7.9°; tooth 33: 7.8–8.3°; tooth 43: 6.7–7.2°) and first premolars (tooth 14: 12.7–13.2°; tooth 24: 13.2–13.3°; tooth 34: 12.3–12.9°; tooth 44: 7.8–8.1°) showed varying degrees of angular deviation, with tooth 13 exhibiting an extremely high value due to one impacted tooth.

Based on the results of independent measurements by two radiologists, the intra- and inter-evaluator reproducibility of manual reconstruction was assessed using the ICC. The intra-evaluator ICC was 0.991 [0.977, 0.996]. The inter-evaluator ICC values for all geometric parameters were above 0.8, indicating good agreement between the two evaluators; Specifically, the ICC values for deviations along the dental arch, midline and plane were 0.830 [0.813–0.845], 0.841 [0.825–0.855] and 0.844 [0.829–0.858], respectively.

### Reconstruction time

The researchers randomly selected 10 cases from each group of the 75 samples, recorded the time required for full dentition segmentation and labiolingual reconstruction, and compared it with manual reconstruction. The results demonstrated a substantial reduction in processing time achieved by the AI model. As summarized in Table 4, the automated process was markedly faster than the manual process across all dentition groups. The overall mean time for automated reconstruction and slicing was 63 ± 4 s, in stark contrast to the manual duration of 5.1 ± 0.2 min. This equates to an average acceleration of approximately 4.85 times.

**Table 4:** Manual and automatic reconstruction time

Group	Manual reconstruction (seconds)	Auto reconstruction (seconds)	Auto reconstruction + Manual Adjustment (seconds)
Normal dentition	313 ± 1	59 ± 1	134 ± 8
Mixed dentition	301 ± 20	69 ± 2	124 ± 23
Crowded dentition	305 ± 7	62 ± 2	118 ± 13
Averages	306 ± 16	63 ± 4	125 ± 16
	*P* < 0.001		

Data are presented as mean ± standard deviation

## Discussion

In the past decade, artificial intelligence (AI) has significantly revolutionized medical image processing, analysis, and diagnosis [26–30]. It has demonstrated exceptional performance across a broad spectrum of tasks, from feature extraction, anatomical structures segmentation, landmark identification and analysis, disease segmentation and diagnosis et al. For panoramic radiograph and CBCT images, deep learning-based automated tooth segmentation is one of the most popular research areas [10, 31, 32]. For instance, Cui [7, 33, 34] 's series of studies achieved high-precision tooth segmentation on CBCT images. Their approach subsequently incorporated distance and confidence-aware cascaded modules for precise segmentation, attaining a Dice coefficient exceeding 98%. Moreover, the cross-center model adaptive dental segmentation framework enhances the model's generalization capability across different clinics [35]. Although high segmentation accuracy and robustness have been attained, the application of these segmentation results remains a critical and under-explored area. Meanwhile, AI-assisted multimodal combining of CBCT with intraoral scanning is another popular research field. By fusing data from both models, automatic registration can be performed to reconstruct high-fidelity 3D tooth models with enhanced accuracy and structural completeness. This approach holds significant potential for multiple clinical scenarios, ranging from preoperative implant planning to periodontal disease diagnosis, thereby streamlining the entire digital dentistry workflow and improving patient-specific treatment outcomes [20, 22, 23, 36]. Currently, studies on using AI tools to assist orthodontic planning based on CBCT images are relatively few. The challenge remains how to make AI applications in CBCT more practical and responsive to real-world clinical needs—for example, automatic full dentition labiolingual reconstruction in orthodontics. This task is considered quite helpful for assessing root-bone relationships and controlling torque, yet the manual reconstruction is highly time-consuming and labor-intensive.

In this study, we developed a DenRec-LL system aiming at automatic full dentition labiolingual reconstruction. The system firstly generated a 2D panoramic image, and a transformer-based segmentation framework, Mask2Former, was employed to achieve tooth segmentation and ID classification from panoramic images. Then through a novel bidirectional 2D-3D integration framework, the method efficiently segments teeth on CBCT-derived panoramic images and maps the results back for 3D-UNet refinement-effectively harnessing the speed of 2D analysis and the precision of 3D volumetric data. Finally, through geometric analysis of tooth axes and arch curves, it enables precise full dentition labiolingual reconstruction. Unlike prior studies focused solely on teeth segmentation, our approach directly converts raw CBCT data into interpretable clinical images. Therefore, by import axial images into our AI platform, a 2D panoramic image and a series of labiolingual reconstructed images for the full dentition will be generated, which will be helpful for dental disease diagnosis and treatment planning.

The clinical significance of this system is more pronounced in the anterior and premolar regions (teeth 15–25 and 35–45), with this assessment focus stemming from clinical and anatomical considerations. Teeth in these areas typically possess a single root or labiolingual double roots, resulting in a labiolingual reconstruction with complete crown-to-apex morphology of the tooth. The value of labiolingual reconstruction images extends far beyond tooth segmentation itself, providing direct diagnostic and planning insights [37–45]. For instance, in orthodontics, the reconstruction images can give a direct labiolingual view of each tooth, thereafter, the clinicians could effectively predict the risk of alveolar bone fenestration and dehiscence during the orthodontic treatment and provides critical reference points for torque control during orthodontic treatment.

In this study, results demonstrated the system's high stability and precision across diverse dentition types, including normal dentition, mixed dentition and crowded dentition. The vast majority of labiolingual reconstruction (93.1%) achieved the highest score (Score 3), and there were no significant differences among the 3 groups, validating the system's robustness. Notably, for normal dentition, there were no teeth scoring 0 or 1 point. For mixed and crowed dentition, there were 9 teeth scored 0–1 point, 5 of them were impacted teeth (Fig. 3C), 1 was because of supernumerary tooth (Fig. 3D). Further analysis by tooth position revealed that teeth with low scores were mainly concentrated in the canines and premolars. Specifically, the average angular deviation for premolars was around 12°, while that for canines was approximately 8°. This is most likely due to the more complex root morphology of the canines and premolars, combined with their location in the transitional zone of the dental arch—where the change in curvature is more abrupt—which may amplify minor axial errors relative to centroids; at the same time, the incidence of anatomical variations such as impacted teeth and supernumerary teeth is higher in these areas. This study also evaluated the geometric deviation and clinical acceptability across three types of dentition. For the AI reconstruction, if the reconstruction plane angular deviation < 5° and reconstruction center point distance deviation < 2.0 mm, we define the reconstruction clinical acceptance. In this study, the clinical acceptance rate exceeded 93% in all three groups, indicating that the AI model has good clinical utility potential. For the teeth with obvious reconstruction deviation, manual adjustment would be necessary. Unlike mainstream segmentation models (e.g. U-Net, TSegNet, cross-center adaptive segmentation models) that focus on mask generation, our DenRec-LL addresses the downstream task of converting segmentation into clinically actionable labiolingual slices [34, 35]. Our evaluation therefore prioritizes slice‑quality metrics (AD, DD) and CAR over segmentation overlap. The results demonstrate that DenRecLL fulfills its clinical purpose.

Moreover, this system shows high efficiency. The entire workflow, encompassing tooth segmentation and full dentition labiolingual reconstruction, takes on average just over 1 min. This stands in stark contrast to the approximately 5 min required for manual operations, representing an average acceleration of approximately 5-fold. This substantial reduction in processing time significantly streamlines clinical workflows and substantially increases its practicality for future routine applications in clinic practice.

**Fig. 5:**
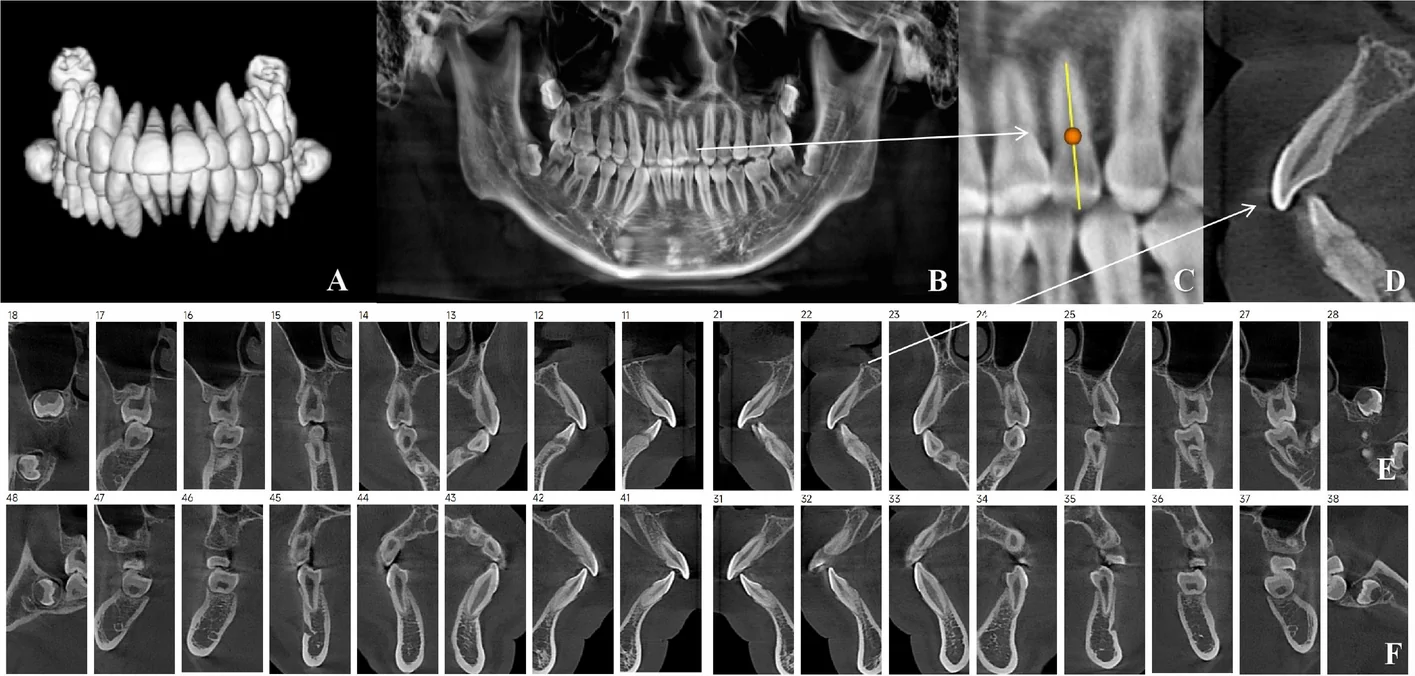
A Representative case of full dentition labiolingual reconstruction. **A** 3D segmentation of the full dentition based on CBCT data. **B** Reconstructed panoramic image. **C** Magnified view of the target tooth (#22), where the yellow line indicates the estimated long axis and the red dot marks the centroid of the tooth. **D** Magnified labiolingual reconstruction view of the target tooth (#22). **E** Labiolingual reconstruction view of the maxillary dentition. **F** Labiolingual reconstruction view of the mandibular dentition

**Fig. 6:**
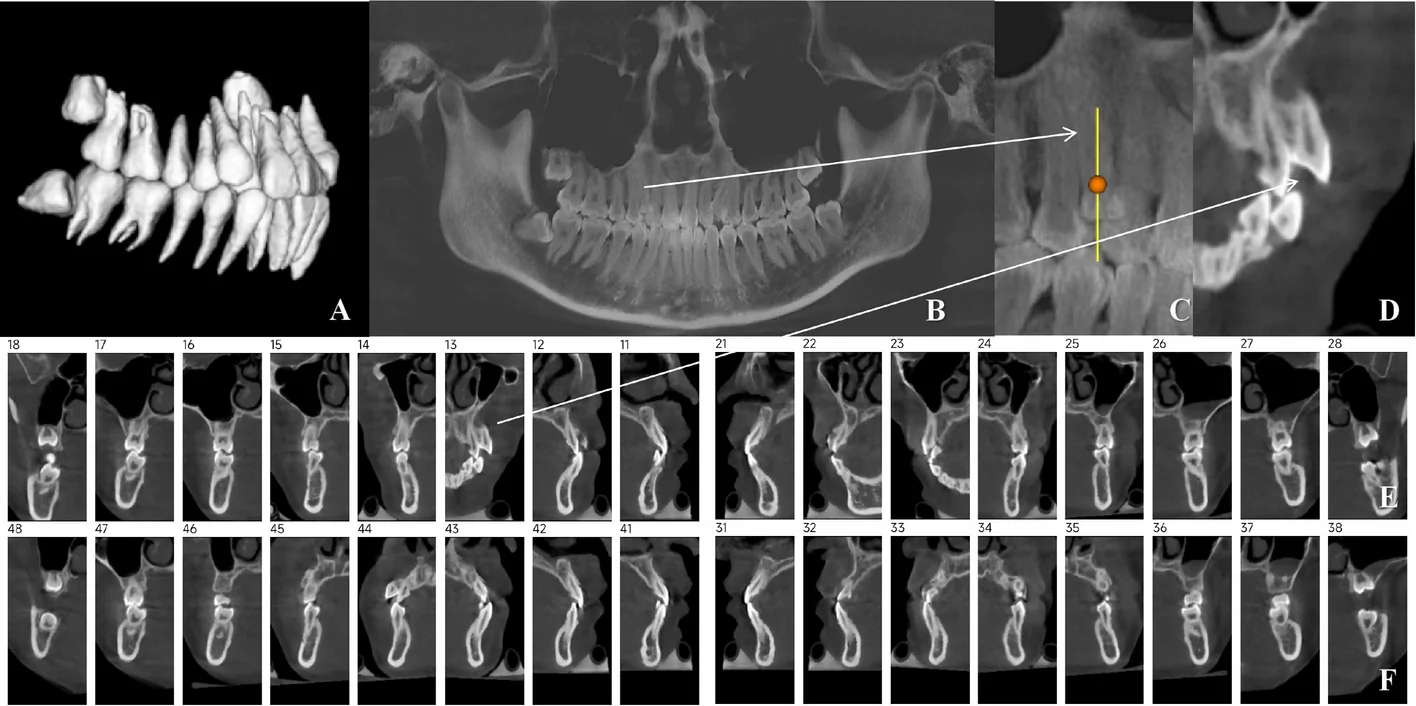
A Representative case of full dentition labiolingual reconstruction. **A** 3D segmentation of the full dentition based on CBCT data. **B** Reconstructed panoramic image. **C** Magnified view of the target tooth (#13) with labial displacement, where the red centroid marker shows a positional deviation due to the ectopic eruption. **D** Magnified labiolingual reconstruction view of the target tooth (#13), showing poor reconstruction. **E** Labiolingual reconstruction view of the maxillary dentition. **F** Labiolingual reconstruction view of the mandibular dentition

Despite the encouraging findings, this study exhibits several limitations that point to valuable directions for future research. Firstly, the existing system is more suitable for reconstruction of anterior teeth and premolars; it has limitations when applied to multi-root molars. This stems from the underlying technical design: our system estimates the long axis of the tooth based on its centroids. For single-root teeth, this centroids approach is capable of generating a clear long axis of tooth. However, for teeth such as maxillary molars with three roots, the centroids approach is defined as the center of the three roots, therefore always resulting in incomplete roots reconstruction (Figs. 2, 5, 6). In future, we will improve this model to make it more suitable for multi-root molars. Secondly, the overall error rate approximately 7% suggests that further refinement of our model is still required before routine clinical implementation. The observed concentration of errors in mixed and crowded dentitions highlights the need for targeted model optimization, particularly by including more cases with specific tooth anomalies (e.g., impacted and supernumerary teeth) in the training cohort to improve the system's generalizability to these clinically challenging cases. Thirdly, the relatively limited sample size necessitates the incorporation of multi-center, multi-device data to enhance the model's robustness. Furthermore, inclusion of additional specific cases (e.g., edentulism) and anomalies (e.g., craniofacial deformities, extensive dental restorations) is required to further validate the system’s performance.

## Conclusion

In summary, we developed a novel and efficient AI system for full dentition labiolingual reconstruction. External testing encompassing normal, mixed, and crowded dentition validated the system's high accuracy, robustness and efficiency.

## Data Availability

The datasets generated and analyzed during the current study are not publicly available due to ethical considerations and the need to protect participant privacy. However, the materials used in this study are available from the corresponding author upon reasonable request.
